# Overview of multifunctional cysteinyl cathepsins in atherosclerosis-based cardiovascular disease: from insights into molecular functions to clinical implications

**DOI:** 10.1186/s13578-023-01040-4

**Published:** 2023-05-19

**Authors:** Xian Wu Cheng, Megumi Narisawa, Hailong Wang, Limei Piao

**Affiliations:** 1 Department of Cardiology and Hypertension, Yanbian University Hospital, 1327 Juzijie, Yanjin, Jilin 133000 People’s Republic of China; 2 Jilin Provincial Key Laboratory of Stress and Cardiovascular Disease, Yanbian University Hospital, Yanjin, 133000 Jilin People’s Republic of China; 3 Department of Cardiology, Nagoya University Graduate School of Medicine, Nagoya, Aichiken 4668550 Japan; 4 Department of Cardiology and Hypertension, Jilin Provincial Key Laboratory of Stress and Cardiovascular Disease, Yanbian University Hospital, 1327 Juzijie, Yanji, Jilin PR. 133000 China

**Keywords:** Vascular remodeling, Protease, Extracellular, Imaging, Biomarker

## Abstract

Cysteinyl cathepsins (CTSs) are widely known to have a proteolysis function that mediates recycling of unwanted proteins in endosomes and lysosomes, and investigation of CTSs has greatly improved with advances in live-imaging techniques both in vivo and in vitro, leading to three key findings. (1) CTSs are relocated from the lysosomes to other cellular spaces (i.e., cytosol, nucleus, nuclear membrane, plasma membrane, and extracellular milieu). (2) In addition to acidic cellular compartments, CTSs also exert biological activity in neutral environments. (3) CTSs also exert multiple nontraditional functions in, for example, extracellular matrix metabolism, cell signaling transduction, protein processing/trafficking, and cellular events. Various stimuli regulate the expression and activities of CTSs in vivo and vitro—e.g., inflammatory cytokines, oxidative stress, neurohormones, and growth factors. Accumulating evidence has confirmed the participation of CTSs in vascular diseases characterized by atherosclerosis, plaque rupture, thrombosis, calcification, aneurysm, restenosis/in-stent-restenosis, and neovasel formation. Circulating and tissue CTSs are promising as biomarkers and as a diagnostic imaging tool in patients with atherosclerosis-based cardiovascular disease (ACVD), and pharmacological interventions with their specific and non-specific inhibitors, and cardiovascular drugs might have potential for the therapeutic targeting of CTSs in animals. This review focuses on the update findings on CTS biology and the involvement of CTSs in the initiation and progression of ACVD and discusses the potential use of CTSs as biomarkers and small-molecule targets to prevent deleterious nontraditional functions in ACVD.

**Supplementary Information:**

The online version contains supplementary material available at 10.1186/s13578-023-01040-4.

## Introduction

Atherosclerosis-based cardiovascular diseases (ACVDs) are high-mortality diseases with a high incidence worldwide. The initiation and progression of various ACVDs are characterized by harmful cellular and molecular changes, including oxidative stress, inflammatory actions, non-resident inflammatory and immune cell infiltration, extracellular matrix (ECM) degradation, cell proliferation, and apoptosis (for review see ref. [1]). Like the serine protease and matrix metalloproteinase (MMP) families, the family of cysteine protease cathepsins (CTSs) has been shown to play an essential role in ACVDs and their complications [2].

Lysosomal CTSs were discovered ~ 70 years ago. At that time, CTSs were recognized to function in intracellular clearance—i.e., in the removal of unwanted proteins in endosomes and lysosomes of cells—and CTSs were thought to be expressed mainly in these two types of cellular organelles [3]. Over the past two decades, laboratory and clinical studies have updated our understanding of lysosomal CTSs in the following four ways. (1) Inducible CTSs are now known to lead to the unraveling of lysosomal and nonlysosomal functions in metabolic and inflammatory diseases such as ACVDs and their complications (i.e., plaque rupture, thrombosis, vasa vasorum, calcification, and restenosis/in-stent-restenosis) [4–9], as well as to angiogenesis and vascularization [10–12]. (2) With the exception of endosomes and lysosomes, CTSs can be secreted into and function in most cellular components—including the extracellular space, plasma membrane, cytosol, nuclear membrane, and nucleus—of inflammatory macrophages and vascular cells, i.e., vascular smooth muscle cells (SMCs), endothelial cells (ECs), and endothelial progenitor cells (EPCs) (for review see ref [13].). (3) Several circulating CTSs and cystatin C have been shown to be effective as prognostic and diagnostic biomarkers for ACVDs [14–19]. (4) Pharmacological CTS inhibition exerts pleiotrophic effects in animals and/or humans [6, 20]. This review focuses on the updated findings regarding the roles of CTSs in ACVDs, highlighting the biology of the members of the CTS family and the significance of CTSs in proteolysis-dependent and -independent molecular mechanisms, including the involvement of Notch-1 signals, receptor activator of nuclear factor-κB (RANK) ligand (RANKL)/signal transducer and activator of transcription 3 (STAT3) signals, the production of angiogenesis-related molecules, histone and histone deacetylase (HADC) modifications, lipid metabolisms, ECM metabolism, inflammation and T-cell activation. Pharmacological inhibition, the diagnosis and prediction of cardiovascular disease and its complications, and bench-to-bedside knowledge are also discussed.

### Biology of the cathepsin family members

#### Brief primer of cysteinyl CTSs: a basic overview

**Fig. 1:**
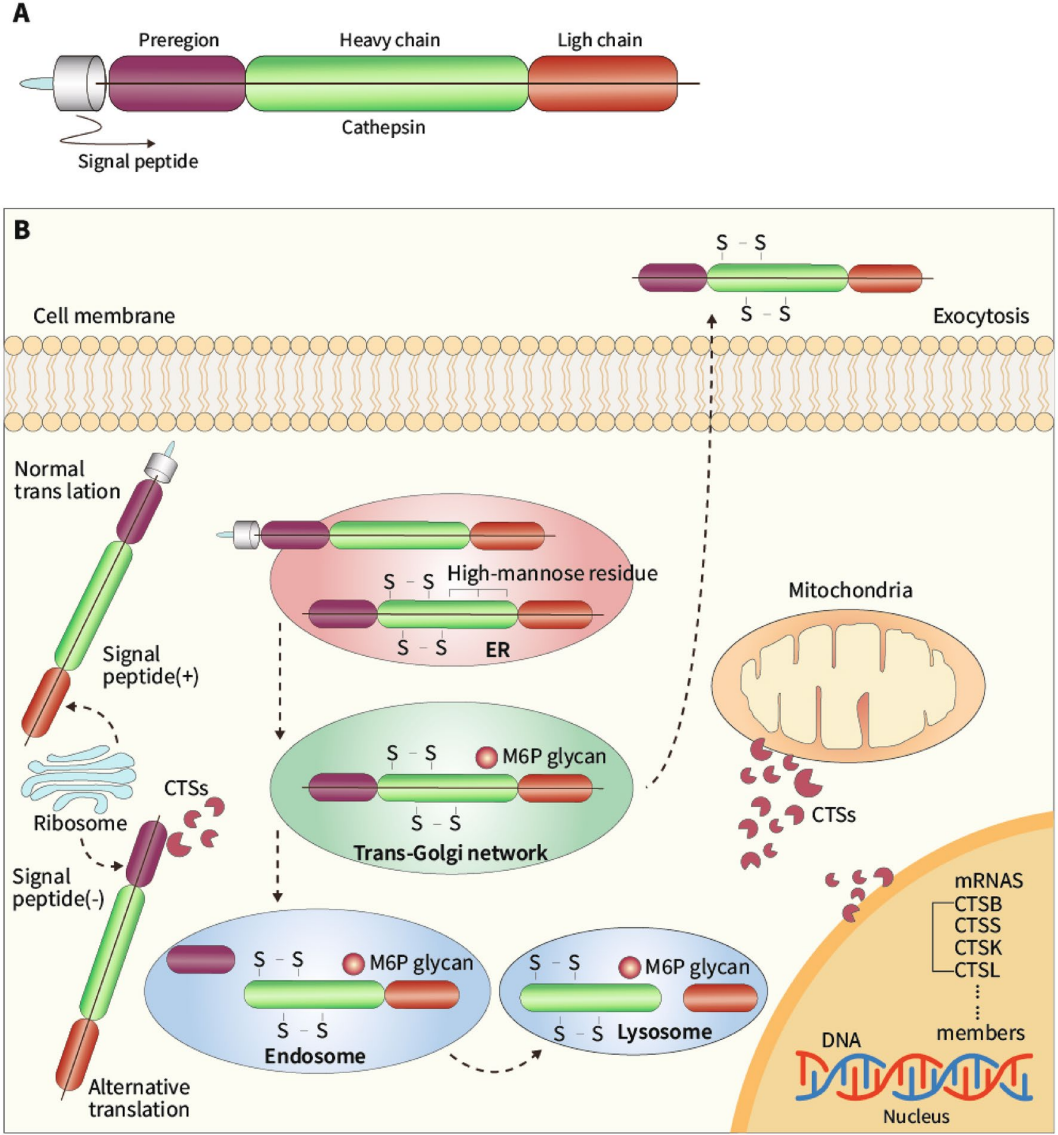
A model of cathepsin (CTS) structure and maturation. A: Each CTS has a signaling peptide, a pre-region, a light chain, and a heavy chain. B: Normal and alternative translations. The general maturation processing of a CTS is as follows. After normal translation, the pro-cathepsin is targeted to the endoplasmic reticulum (ER) for the cleaving and folding of the signaling peptide; to the trans-Golgi network (TGN) for the phosphorylation of mannose-6 residue (M6p); to the early endosome for acidification; to the endosome for the removal of the pre-region; to the lysosome for further activation (light and heavy chains); and then is finally subjected to Ca2+-dependent organelle blending and secretion into extracellular spaces. Some of the CTSs are released by an exocytosis process without proceeding to the M6p form. In the alternative translation, alternative splicing and skipping-related translation generates a CTS without a signal peptide, which moves to the cytoplasm, nucleus, and mitochondria, leading to apoptosis and proliferation

Molecular research has revealed 11 cysteinyl CTSs in humans (CTSK, CTSS, CTSB, CTSL, CTSF, CTSH, CTSC, CTSO, CTSX, CTSW, and CTSZ). Only 9 of these (CTSS, CTSK, CTSB, CTSL, CTSC, CTSF, CTSV, CTSV, and CTSZ) have been confirmed to be linked to the initiation and progression of ACVDs [21]. Similar to the MMPs, these CTSs are generally regulated at three levels: via their genes, activation, and activity [22]. Endogenous cathepsin inhibitors are generally classified as type I, II or III cystatins based on their molecular structures. Cystatin C, a type II cystatin, plays a role in ACVDs and their complications [22]. As documented in an early review literature, most of these CTSs are relatively small proteins with M_r_ values in the range of 20,000 and 35,000 [22]. The primary structure of any CTS has four parts: a heavy chain, light chain, proregion, and signaling peptide (Fig. 1A). This structure is transformed to promote the maturation of CTSs. The transformation takes place in three steps: synthesis in the endoplasmic reticulum, acidification in the endosome, and final activation (Fig. 1B). Because the CTSs have specific binding sites, they play different biological roles, including roles in antigen transfer, signal transduction, proliferation, and apoptosis [4, 23–25].

#### CTS maturation, secretion, activity, and regulation

*Maturation/activation*. CTSs are synthesized as pro-CTSs bearing an N-terminal signaling peptide that guides them into the trans-Golgi network (TGN) [26]. Subsequently, the transport vesicles assist in the delivery of CTSs from the TGN to early and late endosomes for degradation [27]. In the TGN, most CTSs obtain mannose-6-phosphate via the Golgi complex and are targeted by mannose-6-phosphate receptor to promote maturation (for review see ref [13].). Several lines of evidence suggest that lysosomes are vesicles that store matured CTSs and transfer them to the late endosomes when needed [28]. Several proteases (e.g., neutrophil elastase, pepsin, CTSD and various other CTSs) have been shown to accomplish this CTS activation [29, 30]. Complete or partial cleavage of the preregion during maturation influences the folding and stability of the proteases [31]. Thus, limited maturation is an essential step in managing the activity of CTSs.

*Secretion*. Based on the results of experimental studies [32, 33], it has been speculated that CTSs activated, late endosomes and lysosomal CTSs may be secreted into the cytosol via a Ca2^+^-dependent integration of both organelles with the plasma membrane. CTS secretion depends on vacuolar exocytosis with or without the lysosomes. CTSS has been shown to move from the lysosomes to the extracellular space, cell surface, cytosol, and nucleus to act under pathophysiological conditions. The processes from CTS synthesis to secretion are represented schematically in Fig. 1B.

*Proteolytic activity*. Both endosomes and lysosomes are known to provide CTSs with the optimum acidic pH for their activity [3]. CSTK and CTSS can degrade main ECM components (collagen and elastin) both in vivo and in vitro [34–36]. These findings have raised questions regarding how CTSs are released into the extracellular spaces (where the pH is neutral) and how they exert their collagenolytic and elastinolytic activities. In 2000, Punturieri et al. showed that human macrophages form a localized acidic environment in a zone of contact that blocks the neighboring extracellular milieu [37]. They described the use of bafilomycin (an inhibitor of extracellular acidification) and noted that it blocked the elastolytic activity of macrophages, and that an acidic extracellular milieu was created by elevated lysosomal vacuole-type H^+^-ATPase. This finding provides a reasonable explanation for the mechanism by which CTSs degrade the ECM. Later studies demonstrated that CTSs possess multiple novel proteolytic functions, including neuropeptide biosynthesis, prohormone processing, receptor activation, activation and/or release of cytokines, histone H3 processing, and inactivation of growth factors (for review see ref [2]) and other proteases (such as dipeptidyl peptidase-4) in vivo and in vitro.

*Expression regulation*. In 2016, Stellos et al. demonstrated that adenosine-inosine RNA editing can change the content of CTSS in atherosclerosis [38], heralding a new era of genetic editing to change the content of CTSs. Recently, an increased RNA editing rate of Alu elements in CTSS 3′ UTR was shown to be associated with elevated CTSS gene expression in systemic sclerosis patients and rheumatoid arthritis patients [39, 40]. In vascular remodeling, the expression of a CTS is affected by cells and/or biological and chemical factors. The regulation of CTS expression in vascular cells and inflammatory cells is presented in Additional file 1: Table S1. It is well known that many stimuli (cytokines, hormones, growth factors, oxidative stress, oscillatory shear stress, etc.) modulate specific CTS expressions of vascular cells, inflammatory cells, and immune cells in inflammatory and metabolic disorders including ACVDs (Additional file 1: Table S1). For example, five members of the CTS family (CTSL, CTSS, CTSK, CTSB, and CTSD) and cystatin C were shown to be expressed in cultured ECs and/or EPCs in response to cytokines, i.e., stem cell factor, macrophage colony-stimulating factor, interferon-gamma (IFN-γ), stromal-derived factor-1, interleukin-1beta (IL-1β), granulocyte-colony stimulating factor, and tumor necrosis factor-alpha (TNF-α), and in response to oxidized low-density lipoprotein (ox-LDL), reactive oxygen species (ROS), growth factors (basic-fibroblast growth factor [bFGF] and vascular endothelial growth factor-A [VEGF-A]), and shear stress [41–43]. In macrophages, the expressions of CTSs (CTSS, CTSL, CTSK, CTSB, CTSF, and CTSV) were sensitive to ox-LDL, IL-3, TNF-α, and angiotensin II [44]. Unlike in the case of macrophages, there is little information about the relationship between neutrophils and CTS expressions. Among the CTS family members, CTSB, CTSL, CTSK, and CTSS play crucial roles in the transmigration and proliferation of vascular SMCs co-stimulated by IFN-γ and TNF-α both in vivo and in vitro [34, 35].

### CTSs in ACVDs and their complications and neovascularization

The pathogenesis of AVD is substantially implicated in the proteolysis of the vascular wall ECM proteins. Several proteases participate in this process (serine proteases, cysteinyl CTSs, and MMPs). MMPs, which were first described as zinc-dependent proteases, mainly target and cleave extracellular proteins. During the past 20 years, however, the intracellular roles of MMPs have been uncovered; research on this new avenue of MMP biology is summarized in a recent comprehensive review [45]. Thus, the following sections document the implications of CTSs in ACVDs and their complications as well as in vascular regeneration.

#### Atherosclerotic lesions and restenosis

**Fig. 2:**
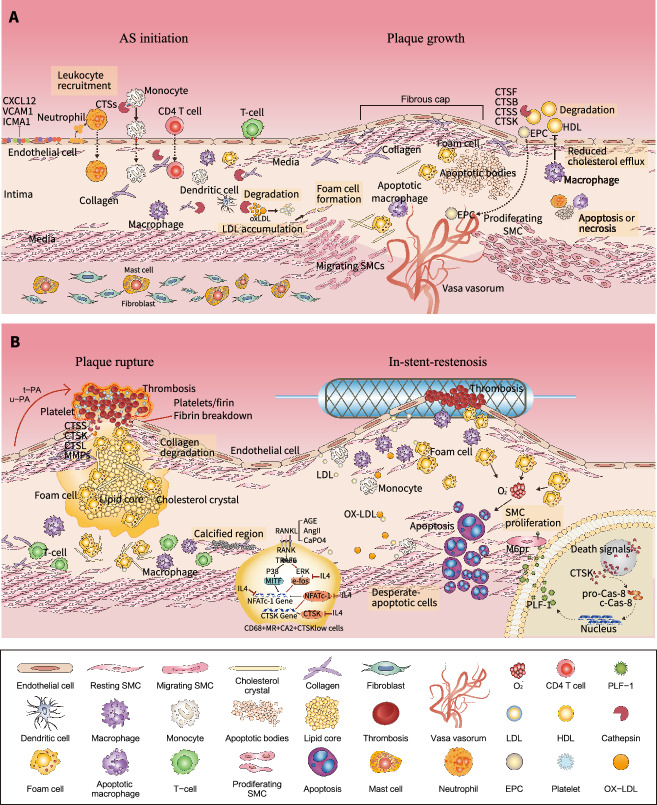
The roles of cathepsins in atherosclerosis (AS). The phases of AS initiation and its complications are shown. Upper panel: Cross-section of a plaque formation. The image at left part of the panel depicts the AS initiation stage: cathepsins can modulate the productions of chemokines such as CXC-chemokine ligand 12 (CXCL12), intracellular adhesion molecule-1 (ICAM-1), and vascular cell adhesion molecular-1 (VCAM-1), which triggers the recruitment of inflammatory cells (neutrophils and macrophages), immune cells (T cells) and endothelial progenitor cells (EPC), adhesion, transmigration, antigen presentation/activation, and proliferation and/or differentiation in the vascular wall. The image at right part of the panel shows plaque containing abundant macrophage foam cells, lipids and necrotic debris, hemmed by a fibrous cap comprised of a collagen-rich matrix. Cathepsins participate in multiple events, including collagen degradation and elastin fragmentation, EPC and endothelial cell migration and vaso vasorum formation, oxidative low-density lipoprotein (OxLDL) degradation to aggregation and accumulation, high density lipoprotein (HDL) degradation to cholesterol efflux reduction, macrophage and smooth muscle cell (SMC)-derived foam cell formation, SMC transmigration and proliferation, and vascular and inflammatory cell apoptosis. Lower panel: Cross-section of plaque rupture, thrombosis, and stent-in-restenosis. As shown at the left, rupture of the fibrosis cap by CVD-related cell-derived cathepsins takes place at the endothelial denudation phase, which triggers the exposure of the highly thrombogenic necrotic core, leading to platelet activation and aggregation and thrombosis and the triggering of a cascade of proteolytic processes such as the releases of CTSG, urokinase plasminogen activator (u-PA) and tissue plasminogen activator (t-PA) by vascular disease cells, facilitating fibrin degradation and lysis of the thrombus. As shown in the middle part of depicting calcification, several factors (AGE, AngII, and CaPO4) trigger the expression of nuclear factor of activated T cells c-1 (NFATc-1) through modulation of either a RANKL/RANK-mediated ERK/e-fos or p38MAPK signaling pathway, which induces calcium mineralization that mediates CTSK in CD68+ MR+ CA2+ CTSKlow cells (called “dysfunctional osteoclast-like cells”). As shown at the right part of depicting stent-in-restenosis, CTSK triggers oxidative stress-induced apoptosis via pro-caspase-8 (pro-Cas-8) maturation, which produces proliferin-1 to stimulate media smooth-muscle repopulation and the development of injury-related hyperplasia/stent-in-restenosis in humans and animals via the activation of a mannose-6-phosphate receptor (M6PR) signaling cascade. CA2: carbonic anhydrase type II; CTS: cathepsin; ERK: extracellular signal-regulated kinase; GM-AGE: glycoladehyde-modified advanced glycation endproducts; IL: interleukin; MITF: microphthalmia transcription factor; p38MAPK: p38 mitogen-activated protein kinase; RANK: receptor of activator of nuclear factor-κB; RANKL: RANK ligand

*Atherosclerosis*. In the mid-1990s, the results of an in vitro experiment demonstrated by vital fluorescence and active-site labeling methods that human monocyte/macrophage-derived CTSL, CTSS, and CTSB possess elastolytic activity [36]. CTSK and CTSS were the first cysteinyl CTSs that were observed to show elevated protein levels in human atherosclerotic aneurysm lesions in which cystatin C was reduced [46]. CTSS and cystatin C had similar expression patterns in neointima lesions formed in response to balloon injury in rats [7]. Although vascular cells and macrophages express negligible amounts of CTSS, CTSK, and CTSL, the expressions of these enzymes were augmented under stimulation with inflammatory cytokines [46]. The ability of macrophages and SMCs to use CTSs to disrupt collagen and elastin supports the idea that these enzymes play essential roles in cardiovascular remodeling in animals and humans. This concept is further supported by the established genetic model studies of apolipoprotein E knockout (*ApoE*^−/−^) and low-density lipoprotein receptor knockout (*Ldlr*^−/−^) mice, in which CTSS^−/−^ [47], CTSK^−/−^ [48], and CTSL^−/−^ [49] mitigated plaque formation and preserved vascular elastin and collagen disruption. The roles of CTSs in atherosclerosis were covered by a recent comprehensive article [21] and thus will not be described in detail here. The roles of CTSs in atherosclerotic lesion formation, plaque growth, and their complications are represented schematically in Fig. 2.

*In-stent-restenosis*. In-stent-restenosis, which is characterized by intimal hyperplasia, affects the long-term prognosis of patients after percutaneous coronary intervention. The transmigration of SMCs from the media to the intima is a key step in in-stent-restenosis. In a rabbit balloon-injury model, increased CTSS and CTSK and decreased cystatin C were found to promote the development of neointima [7]. In a ligation model, CTSK was observed to play an essential role in experimental neointimal formation under chronic stress, possibly by reducing TLR-2/4-mediated inflammation and VSMC proliferation [34]. Similar to CTSK, CTSS and CTSL can also promote neointimal hyperplasia via the modulation of medial SMC migration and proliferation, and this can be partially prevented by reducing the expressions and activities of both CTSS and CTSL with the use of specific inhibitors or gene knockout [6, 50]. A clinical investigation revealed that an increased level of plasma CTSS could be a predictor of in-stent-restenosis at 6 months after a percutaneous transluminal angioplasty [51]. Taken together, these findings suggest that CTSs exert actions in in-stent-restenosis, mainly by inducing inflammation and promoting the migration and proliferation of SMCs, as discussed in the below sections on the individual functions of CTSs.

#### Plaque rupture, thrombosis, and calcification

It is widely recognized that the rupture of a vulnerable arterial plaque and the related thrombosis can trigger an acute myocardial infarction and stroke [52]. A vulnerable atherosclerotic plaque is composed of a thin fibrous cap, lipid-rich necrotic core, calcified cholesterol crystal, infiltrated inflammatory and immune cells (macrophages, mast cells, T cells, and B cells), abundant proteolytic enzymes (CTSs, MMPs, and a disintegrin and metalloproteinase), scattered collagen, and disrupted elastin (Fig. 2B, left) [53, 54]. The lack of a dependable animal model over the past two decades has limited understanding of the molecular mechanisms of human plaque rupture and its early prevention, but in 2006 our group developed a simple and useful plaque rupture model using carotid arterial ligation and polyethylene cuff replacement in ApoE^−/−^ mice [55], and this approach was soon applied to the screening of lipid-lowering agents and other drugs [52]. Laboratory and clinical evidence indicates that the SMCs, ECs, and infiltrated macrophages function as major cell sources for the CTSs (CTSS, CTSK, CTSL, and CTSB) in human and animal atherosclerotic lesions [46, 56]. As anticipated, cystatin C^−/−^ lowered the contents of SMCs and collagen and reduced the fibrous cap thickness [57]. Conversely, a deficiency in CTSS in *LDLR*^−/−^ mice fed a high-fat diet resulted in a marked reduction of atherosclerotic lesion formation and prevented elastic laminal disruption [47]. We also previously reported that CTSS^−/−^ rectified harmful changes in plaque (i.e., changes in SMC content, collagen volume, and fibrous cap thickness), leading to a reduction of the numbers of plaque ruptures, whereas cystatin C^−/−^ enhanced these changes. In addition to CTSS, CTSK of macrophage-derived foam cells degraded human atherosclerotic plaque type I collagen and elevated CTSK activity, leading to plaque instability [58]. The disruption of the CTSK gene in apoE^−/−^ mice ameliorated atherogenesis progression and increased plaque fibrosis, although it also accelerated macrophage foam cell formation [48]. We similarly reported that pharmacological inhibitions of CTSS and CTSK had a vascular benefit as in these previously reported, genetically modified mice [59, 60]. In vivo and in vitro studies unveiled an unanticipated feedback of CTSC^−/−^ on T-helper cell differentiation and macrophage activation procedures, whereas CTSC^−/−^ led to a facilitation of M2 and Th2 polarization and the attenuation of plaque instability [61]. These studies thus established that increases in CTSs can trigger plaque vulnerability and rupture (Fig. 2B, left), indicating a potential therapeutic strategy for the management of ischemic ACVD events via the modulation of CTS and MMP activities. It should be noted that features of the ruptured fibrous cap in animals have often been used to study plaque instability [52], but it remains unknown whether these structural characteristics completely mimic human plaque ruptures [62].

*Thrombosis.* Arterial atherosclerotic thrombus formation often involves plaque rupture and results in cessation of blood flow, causing acute myocardial infarction or stroke. Indeed, thrombosis is usually caused by a physically ruptured plaque, and it appears to be associated with a loss of collagen volume in the fibrous cap of the plaques by CTSs- and MMPs-mediated proteolysis [2]. Nonetheless, there have been few investigations of the functions of CTSs in the thrombosis of atherogenic lesions. CTSS was the first member of the CTS family that was proposed to play role in thrombus formation [2]. Diabetes and obesity resulted in increases in the levels of stefin A/cystatin A (known as an inhibitor of CTSB, H, and L) in megakaryocytes and platelets of humans and rats [63]. Stefin A and cystatin A have been shown to be localized primarily at granules and platelet membranes and to be released upon agonist induction and clot formation via an MMP-dependent mechanism [63]. Both stefin A and cystatin A can reduce platelet accumulation on immobilized collagen from the circulating blood, with no influence on platelet aggregation. Both in vivo and in vitro, the inhibition of CTS and the overexpression of stefin A were each shown to exert an anti-thrombosis effect [63], suggesting that stefin A and cystatin A act as critical negative modulators of platelet-mediated thrombosis in humans and animals. On the other hand, it was reported that the CTSA activity of a parietal thrombus of aneurysm was markedly increased compared to blood clot CTSA activity [64]. Using an iron chloride3 (FeCl3)-induced carotid artery thrombosis model, we recently demonstrated that chronic stress accelerated the thrombus formation associated with the induction of CTSS expression and activity [8]. In the same model, we reported that stress accelerated thrombotic action and shortened blood clotting times, accompanied by elevated plasma plasminogen activator inhibitor-1 and von Willebrand factor activities and decreased levels of plasma a disintegrin and metalloproteinase with a thrombospondin type 1 motif, member 13, in CTSS^−/−^ mice [65]. Although these findings all contribute to our understanding of the roles of CTSs in thrombosis in humans and animals, the mechanism by which the upregulation of CTSs influences thrombus formation and whether their pro- and anti-thrombotic properties impact atherosclerotic complications remain largely unknown.

*Calcification*. Vascular calcification often results in serious complications such as plaque rupture and aortic valvular stenosis [66]. Accumulating evidence indicates that vascular and valvular calcification occur via the modulation of complicated processes that are characterized by the expression of osteogenesis-related matrix proteolytic molecules (namely, members of the CTS and MMP families) by lesion macrophage- and SMCs-derived osteoclast-like cells [67]. We discuss the roles of CTSs in calcification in detail below (see the *RANKL/RANK and STAT3 signaling* section).

#### Atherosclerosis-related vasa vasorum

It has become clear that the vasa vasorum promotes atherosclerotic plaque growth and rupture in humans and animals [2]. Experimental studies have shown that MMPs participate in plaque neovessel formation and growth [68], but there is limited information regarding the role of CTSs in vasa vasorum formation. We have demonstrated that the injured carotid arteries contain increased numbers of microvessels accompanied by increased CTSK expression; these changes were rectified by CTSK deletion and CTSK-II treatment [9]. Although many investigations have indicated that CTSs are involved in angiogenesis and vascularization, as discussed above, in vivo studies using inhibitors or genetic mice to define the role of CTSs in the vasa vasorum have been lacking.

#### Aneurysm formation

Aortic aneurysm, one of the critical atherosclerotic complications, is characterized by abundant inflammatory cell infiltration, medial destruction and SMC loss, and collagen and elastin degradation [21]. As in atherogenic lesions, the infiltrated inflammatory/immune cell-derived CTSK and MMPs lead to destructive elastolysis and the recruitment of more inflammatory cells, medial SMC apoptosis, collagen/elastin loss, and vasa vasorum formation, which facilitate aortic dilation [69]. In human and animal aneurysm lesions, CTSs (CTSB, K, L, and S) are highly expressed, whereas cystatin C is lacking [21]. The hallmark of aneurysm pathology is an imbalance between CTSs and their endogenous inhibitors in the vascular wall, which causes extensive ECM degradation and progressive weakening of the aortic wall [21]. Genetic animal studies of CTSs established these roles of CTSs and their molecular mechanisms during atherosclerotic aneurysm formation [69, 70].

#### Neovascularization

Angiogenesis and vascularization are two of the important pathological events in ischemic ACVD. In a monocyte-endothelial co-culture, TNF-α maximally upregulated CTSK and CTSV activities compared to either cell type alone, in a JNK axis-dependent manner. In addition to these inflammatory cytokines, bFGF-2 and VEGF-A also induced CTSS expression and activity [41]. In a study on femoral artery ligation-induced angiogenesis of young CTSS^−/−^ mice and CTSS^+/+^ mice treated with the CTSS-specific synthetic inhibitor Z-FL-COCHO, we observed poor capillary formation and blood flow recovery in the ischemic muscles, which were associated with decreased levels of VEGF protein [11]. We also found that the ischemic muscles contained lower levels of insulin-like growth factor-1 receptor substrate-2 and peroxisome proliferator-activated receptor-γ, which were mediated by the inactivation of the Akt-mammalian target of rapamycin and p38-mitogen-activated protein kinase-extracellular signal-regulated kinase1/2 pathways. Moreover, in ECs, silencing or overexpression of CTSS respectively enhanced or reduced IRS-2/PPAR-γ levels and their regulating signaling [11]. Both of these interventions also impaired EC angiogenic actions induced by VEGF and FGF, i.e., migration, invasion, proliferation, and microtube formation [41]. Thus, these findings suggested that EC-derived CTSs can activate angiogenic actions via the mediation of targeted growth factor/receptor signaling activations.

*Vascularization*. Many studies have also shown cathepsin activity in vascularization. In 2005, Urbich and colleagues demonstrated that CTSL was more highly expressed in bone-marrow EPCs than in matured ECs in mice [12]. In a mouse model of hindlimb ischemia, infused CTSL^+/+^ EPC greatly improved neovascularization, whereas infused CTSL^−/−^ EPCs provided limited benefit. The same group reported that high glucose greatly reduced the CTSL expression and CTSL-induced gelatinolytic activity and invasion ability of bone marrow-derived EPCs, but did not reduce the expressions and corresponding activities of CTSD or CTSO in this model [12]. This finding was reinforced by their later clinical observation that the EPCs of patients with type 2 diabetes had profoundly reduced CTSL expression and activity compared to the EPCs of healthy subjects [71]. Bone-marrow EPC-derived CTSL is required for choroidal and retinal vascularization [72]. CTSL-knockout mice also exhibited a defective cardiac regeneration capacity, with the decreases in VEGF impairing bone-marrow-derived c-Kit^+^ stem cell mobilization after acute myocardial infarction [73]. Like CTSL, hypoxic/ischemic stress increased the expression of CTSS in bone-marrow EPCs. CTSS^–/–^ resulted in markedly reduced neovascularization accompanied by reduced VEGF levels in EPCs of young and aged mice after ischemia [11, 74], suggesting a role for CTSL and CTSS in bone-marrow EPC-related vascularization that is dependent on VEGF-dependent and -independent signaling pathways. This concept has contributed to a deeper understanding of CTSs and merits further investigations of the paracrine mechanisms underlying ischemia-induced neovascularization in the management of ACVDs. It should be noted that, unlike modifications CTSL, CTSS modifications had no effect on cardiac angiogenesis after acute myocardial infarction [75]. Thus, although most of the CTSs show the same expression patterns in response to hypoxia [11, 12], the exact function of specific CTSs in neovascularization in different animal models and the underlying mechanisms are largely unknown and inconsistent.

### Cathepsin function: mechanisms of action on molecular and cellular levels

#### Molecular levels

**Fig. 3:**
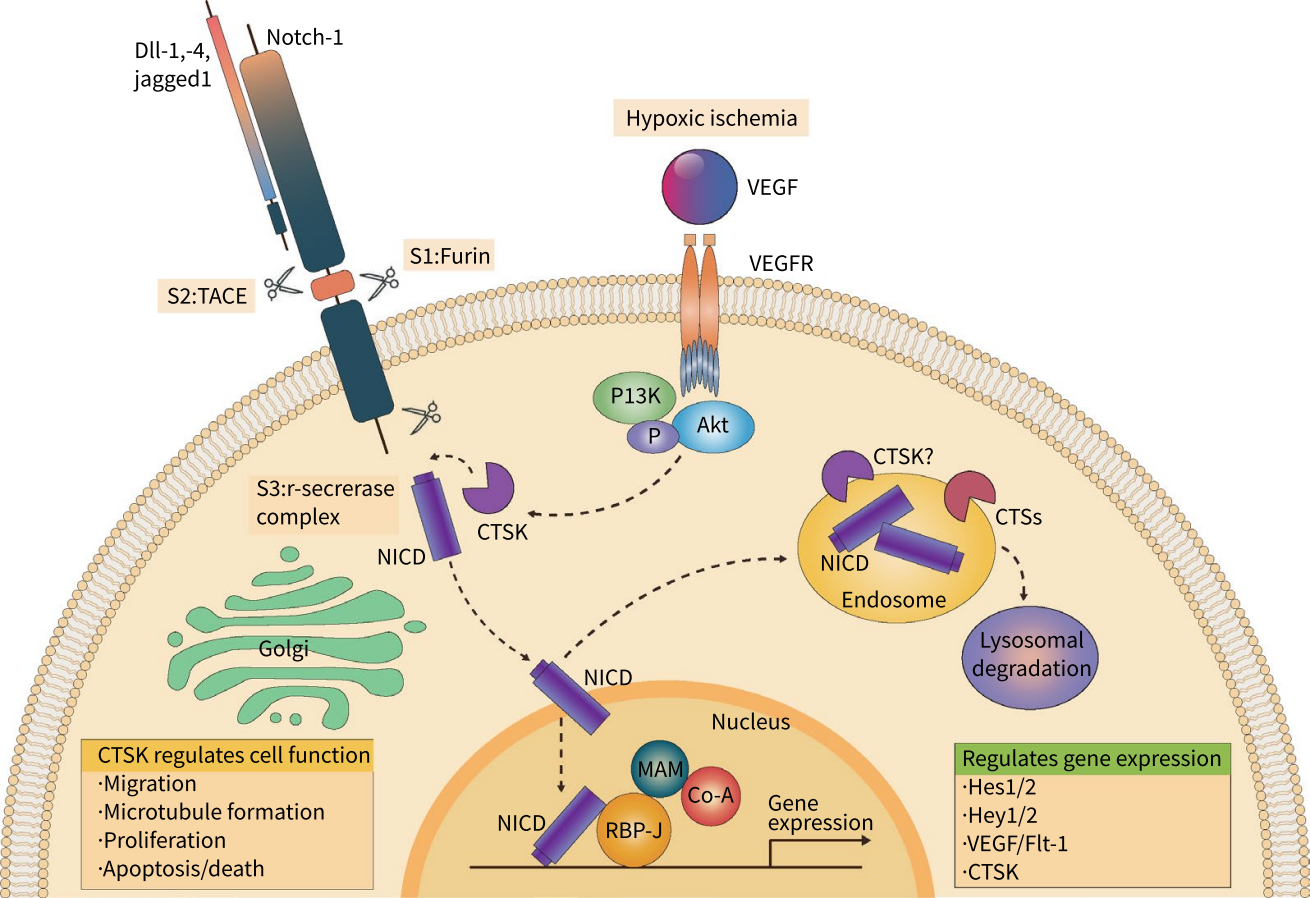
A molecular model of CTSK-mediated Notch-1 activation in endothelial cells. The notch1 receptor is synthesized in precursor forms that are cleaved by the enzymes at the following three sites. S1, by furin-like convertase to generate the mature receptor; S2, by the metalloproteinase tumor necrosis factor α-converting enzyme (TACE, also known as ADAM17); and S3, by γ-secretase or cathepsin K (CTSK) to generate the Notch intracellular domain (NICD). VEGF stimulates CTSK expression by VEGFR-PI3K/Akt signaling activation in endothelial cells. Following CTSK-mediated proteolytic processing, the NICD moves into the nucleus, which triggers downstream angiogenic factor transcription for the modulation of EC migration, microtubule formation, proliferation, and apoptosis/death. The NICD is also targeted to the endosome for degradation. Akt: protein kinase B/Akt; Flt-1: fms-like tyrosine kinase 1; Hes-1: hairy enhancer of split homolog-1; Hey-1: Hes-related repressor protein-1; MAM: master mind; PI3K: phosphatidylinositol 3-kinase; VEGF: vascular endothelial growth factor; VEGFR: VEGF receptor

*Notch-1 signaling*: Endothelial Notch-1 has been shown to be critical for angiogenesis and vasculogenesis [76]. The binding of Notch-1 receptor to Dll or Jag family ligands is cleaved by γ-secretase, and then the Notch-1 intracellular domain (NICD) moves to the nucleus to bind with the RBP-J transcriptional complex to facilitate the transcription of downstream molecule expressions (Hes1/2 and Hey1/2) [76]. In 2014, we reported that hypoxic/ischemic stress resulted in a dramatic increase in both CTSK activity and NICD levels in the ischemic muscles of mice [10]. CTSK deletion impaired the functional recovery of the ischemic muscles, with decreased levels of pro-angiogenic proteins (cleaved NICD, Hes-1 Hey-1, Hey-2, and VEGF-A) in the ischemic tissues. We observed that, in vitro, CTSK silencing or overexpression yielded the respective alterations in cleaved NICD levels and the downstream signaling of NICD in ECs and EPCs, whereas the activity of other targeted CTS family members (CTSB, CTSS, and CTSL) had no such effect. These findings provided the first evidence and mechanistic interpretation of a unique role of CTSK, i.e., contributing to NICD processing and signaling activation (Fig. 3). In the same study, we observed that CTSK^−/−^ mice had defective EC transmigration/invasion, proliferation, and microtube sprouting as well as defects in the mobilization and angiogenic actions of CD31^+^/c-Kit^+^ EPCs. Moreover, cell therapy with bone marrow-derived EPCs of CTSK^+/+^ mice restored the reduced neovascularization in CTSK^–/–^ mice. Thus, the augmentation of CTSK-mediated Notch1 activation (considered a "good guy" in terms of ischemic ACVD) by chemical and/or engineering approaches may have potential utility for improving the efficacy of therapeutic neovascularization in the treatment of ischemic ACVD.

*Pro- and anti-angiogenic molecule signaling*: Our data and those of colleagues Shi et al. revealed that even under normal growth factor conditions, the activity of CTSS still modulates microvessel development in response to wound injury [77]. Those mechanistic investigations indicated a role for CTSS in ECM protein metabolism-related pro- and anti-angiogenic molecule productions during the angiogenesis process [77]. In a subsequent study of a pancreatic islet cell carcinoma model, Wang et al. performed an in-depth analysis of paired CTSS^–/–^ mice and cystatin C^−/−^ mice and observed a critical role of CTSS in angiogenesis via a proteolysis-mediated production of proangiogenic-γ2 (laminin-5-derived) fragments and the elimination of anti-angiogenic canstatin and arresten (collagen-derived) [77]. In HUVECs, the silencing of CTSL and CTSS, but not CTSB, decreased endostatin production (< 22 kDa) [78], suggesting that endostatin-derived angiostatic peptides represent molecular links between CTSs and aminopeptidase N/CD13 in the inactivation of angiogenesis.

*HDAC signaling*: HDACs modified by the removal of the lysine residues from histone and non-histone proteins modulate epigenetic gene expression (for review see ref. [79]). HDACs play an essential role in the pathogenesis of proliferative diseases, including restenosis and atherosclerosis [5]. Adams-Cioaba et al. revealed the interaction of CTSL and histone H3 and described the structure of the CTSL-substrate complex in 2011 [80]. In mouse embryonic stem cells, CTSL was earlier shown to modulate differentiation by a proteolytic processing of histone H3 [81]. In 2016, we revealed that CTSS activity modulates injury-induced neointimal hyperplasia via TLR2-mediated p38MAPK/Akt-HDAC6 signaling activation [5]. Later in vivo and in vitro experiments demonstrated that the failing heart has an increase in CTSS-induced cleaving of the N-terminal fragment of HDAC4, and HDAC4 overexpression prevents cardiac remodeling and dysfunction through a reduction of Nr4a1 expression and an NR4A1-dependent activation of the hexosamine biosynthetic pathway [82]. These findings raised a clinical possibility: it might be possible to develop small molecule-targeted CTS-mediated histone and HDAC modification for the management of proliferative ACVD and its complications.

*RANKL/RANK and STAT3 signaling*: CTSK is the best-studied CTS in vascular extracellular vesicle microcalcification [83]. In an osteoclast-lineage cell line, actin-binding molecular coronin 1A negatively regulated lysosomal fusion and CTSK leakage, which respectively control bone resorption and extracellular calcium accumulation [84]. The specific deletion of leucocyte CTSK caused phenotypic alterations in bone mineral density and atherosclerotic lesion components (elastin fragmentation, collagen volume, macrophage/calcification content, and apoptotic area) in LDLr^−/−^ mice fed a high-fat diet [85]. Moreover, the calcified aortic aneurysm tissues of humans and those of murine aneurysm models induced by AngII infusion or CaCL_2_-soaked gauze contained abundant expressions of CTSK, MMP-9, and tartrate-resistant acid phosphatase (TRAP) that were mainly co-localized in CD11^+^/TRAP^+^ osteoclast-like cells [83]. Strikingly, glycolaldehyde-modified advanced glycation end products suppressed the numbers of TRAP^+^ cells and the expression levels of nuclear factor of activated T cell c-1 (NFATc-1) and the CTSK gene and upregulated NF-κB phosphorylation and IL-10 expression, which mitigated human CD14^+^ cell differentiation into osteoclasts and calcium accumulation; these beneficial effects were abrogated by an IL-4 neutralizing antibody [85], indicating that IL-4 influences osteoclast differentiation and plaque calcification via the modulation of NF-κB-dependent IL-10/CTSK production in diabetic patients. In 2017, Chinetti-Gbaguidi and colleagues confirmed that the calcified areas of human atherosclerotic plaque were occupied mainly by CD68^+^/M6pR (mannose-6-phosphate receptor)^+^ double-positive alternative macrophages [86]. In the same paper, IL-4 was shown to induce macrophage differentiation into osteoclast-like cells that exhibited high-level expression of the M6pR gene and the osteoclast marker carbonic anhydrase type 2 (CA2) gene. IL-4-polarized macrophages showed increased expressions of RANK and trimethylation of its promoter, transcriptional repression marker histone 3 lysine 27, but a defective RANK ligand (RANKL) downstream of ERK-1/-2 phosphorylation and c-fos expression, and this defect led to the impairment of RANKL-induced osteoclast TRAP activity and transcriptional regulator NFATc-1 expression, which in turn triggered defective CTSK expression and activity in CD68^+^/M6pR^+^/CA2^+^ osteoclast-like cells [86]. Together with the clinical data of oxidative stress-related cardiovascular calcification in chronic kidney disease patients [87], these findings indicate that the upregulation of osteoclast-like cell-derived CTSK expression and activity by RANKL/RNAK-p38MAPK or -Erk-1/-2 signaling activation can represent a common mechanism in atherosclerotic plaque calcification (Fig. 2B, middle), implying that CTSK might be a unique molecular target for the management of inflammatory and metabolic ACVD and high-risk calcification.

Additionally, CTSS not only exhibits an expression pattern that is similar to that of MMP-9; it also plays a role in matrix degradation-mediated microcalcification. In addition to the specific macrophage MMP-9 gene modification that accelerated plaque calcification in transgenic rabbits [88], whole-body elastic CTSS deletion can affect atherogenic diet-induced aortic valvular microcalcification in mice with chronic renal disease [89]. CTSS silencing also resulted in an imbalance between adipose and osteoclast differentiation, an elevated level of the circulating bone formation biomarker osteocalcin, and an increase in type 1 procollagen amino-terminal-propeptide along with an enhanced expression of osteoblast-specific mRNAs and changes in the bone turnover and microarchitecture in mice [90]. Several experiments revealed that an H_2_S donor, NaHS, ameliorated high glucose-induced human aortic SMC calcification by decreasing calcium deposition, STAT3 activation, and CTSS expression and activity by increased elastin protein [91]. The inhibition (genetic or pharmacological) and the overexpression of STAT3 (but not mutant C259S) positively and negatively regulated CTSS expression, respectively, which triggered elastin alterations; both interventions of CTSS had the anticipated effect on elastin loss and calcification [91], suggesting that increased CTSS activity activates vascular and valvular calcification, which use the STAT3 signaling pathway. On the other hand, it is now clear that vascular and valve pathological calcification require several other types of signaling, including transforming growth factor-β1 and Rac2/IL-1β signaling pathways [92, 93]. There have been limited studies on the role of CTS family members in the initiation and progression of microcalcification via these known and unknown signaling pathways [21]. Further investigations are necessary to explore this issue, and the results may contribute to the management of ACVD patients with calcification lesions.

*Cholesterol uptake, modification and efflux*: Lipoprotein uptake and modification by atherosclerotic lesion cells, mainly macrophages and SMCs, are essential pathological steps in atherosclerotic plaque formation [21]. Here we summarize the roles of CTSs in lipid metabolism as follows. (1) *The uptake of modified LDL*: CTSK^−/−^ of bone-marrow-derived macrophages showed an increased uptake of modified LDL associated with increased CD36 and caveolins [48]. The authors of that study demonstrated that the cholesterol ester storage was increased in the large lysosomal compartments of ApoE^−/−^/CTSK^−/−^ bone marrow-derived macrophages compared to control ApoE^−/−^ macrophages, which suggests that CTSK modulates foam cell formation. (2) *The degradation of modified LDL*: In human aortic SMCs, the inhibition of CTSB activity reduced oxidized LDL by 41% under low-acid conditions [94]. In contrast to the degradation of apolipoprotein B-100 by CTSS and CTSK, CTSF triggers LDL fusion and aggregation and an increase in the binding ability of LDL to proteoglycans, subsequently leading to the accumulation of extracellular lipid droplets [95]. CTSD facilitates lipid metabolism by decreasing the conversion of cholesterol into bile acids in vitro and attenuating excretion of bile acids via the feces in vivo [96]. Other CTSs such as CTSB, CTSL, and CTSL2 cleave ApoB-100, leading to an expansion of LDL particles [97]. These data thus provide evidence that most of the CTSs can modulate lipid accumulation in the extracellular spaces of the cardiovascular wall of atherosclerotic plaques. (3) *Cholesterol efflux*: CTSs also participate in cholesterol efflux. The activities of CTSF and CTSS disturb the high-density lipoprotein-mediated cholesterol efflux from foam cells [98]. Plasma CTSS was identified as exerting the most proteolytic ability on one of the major protein components of high-density lipoproteins, apolipoprotein A-1, in patients with aortic valve stenosis and a rabbit model of aortic valve stenosis [99]. In vitro, CTSS completely degraded lipid-free apolipoprotein A-1, leading to a loss of the ability of apolipoprotein A-1 to facilitate cholesterol efflux. Moreover, the cleavage of apolipoprotein A-1 by human macrophage-derived CTSB at Ser228 is a functionally important post-translational modification that limits its anti-atherogenic properties [100]. Although the degradation potencies of CSTK and CTSF are lower than that of CTSS, both also partially degraded preβ- high-density lipoprotein and apolipoprotein A-1 [98], indicating that the cholesterol efflux reduction by these increased CTS-mediated degradations of cholesterol acceptors with MMPs may be conducive to the preservation of foam cell characteristics in atherosclerotic lesions. Taken together, the above-described findings have clarified the roles of CTSs in LDL uptake, storage, and efflux (Fig. 2A, right). However, one of the most critical questions remains to be answered—namely, whether the upregulations of CTSs in lipid metabolism are atherogenesis-protective or -stimulating. Further laboratory and clinical studies are required to elucidate this issue.

**Fig. 4:**
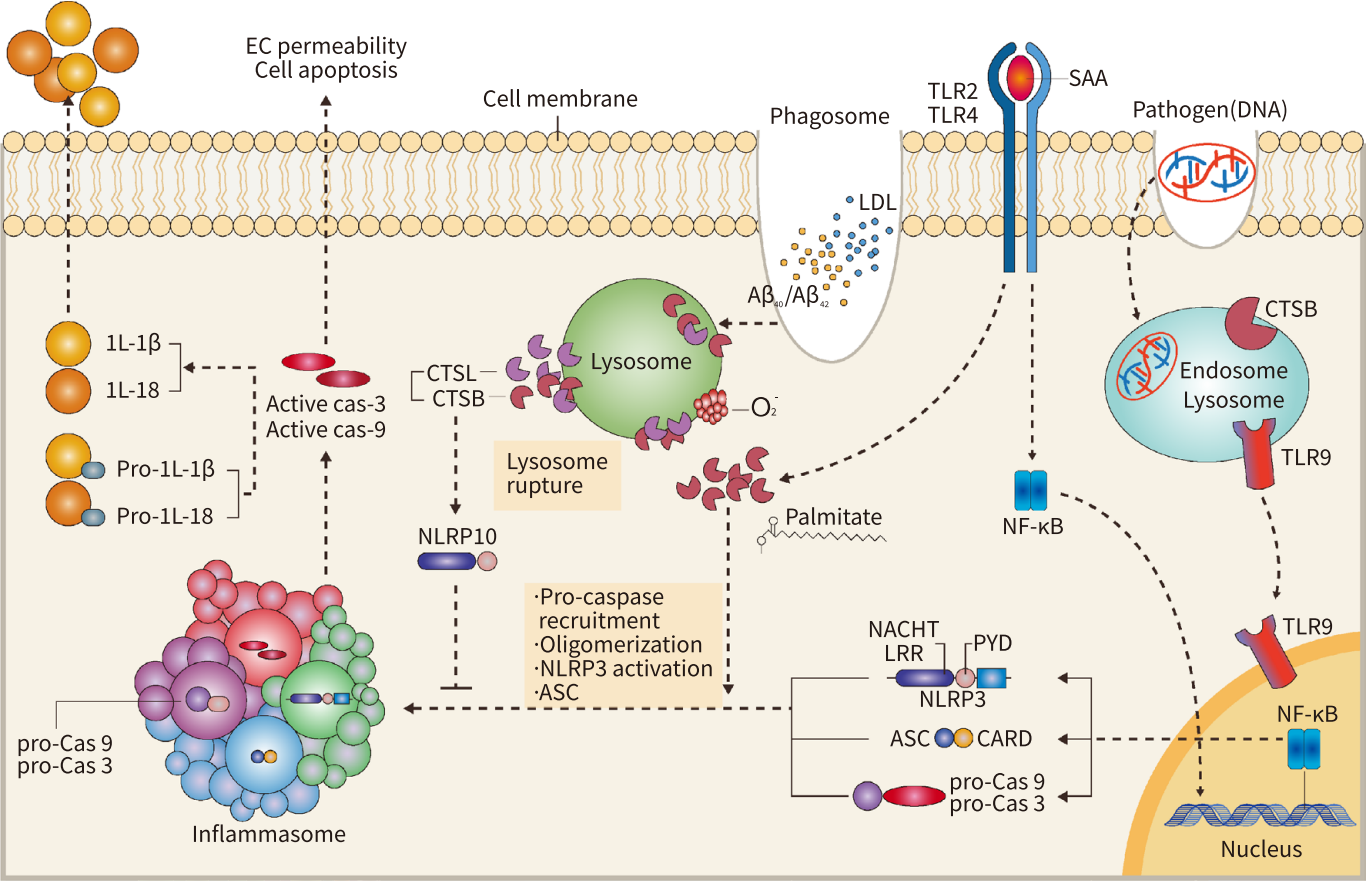
The roles of cathepsins in inflammation response. NLRP3 inflammasome activation occurs in the following ways. (1) Lysosomes ruptured by ROS and phagocytosis-derived molecules (LDL and Aβ40/42) release CTSB and CTSL into the cytoplasm, where they activate NLRP3 containing leucine-rich repeat (LRL), NACHT, and pyrin domain (PYD) domains and induce oligomerization with pro-caspase-3/-9 (pro-cas-3/-99) and apoptosisrelated speck-like protein including a caspase recruitment domain (CARD-ASC) to develop the inflammasome complex. (2) CTSB and CTSL negatively regulate NLRP3 inflammasome activation via the degradation of NLRP10. (3) CTSB can also facilitate the formation of the inflammasome NLRP3 complex induced by the free fatty acid palmitate and toll-like receptor 2/4 (TLR2/4)-mediated blood serum amyloid A (SAA) protein. The activated NLRP3 inflammasome generates the active caspase-3/-9 (cas-3/-9) form that can result in endothelial cell permeability/apoptosis or the activation of pro-interleukin (IL)-1β and pro-IL-18, thereby releasing these mature cytokines into blood. EC: endothelial cell; NLRP3: specific NACHT, LRR, and PYD domains-containing protein 3; NF-κB, nuclear factor-κB

*Inflammation*: It is generally believed that inflammation caused by CTS is mainly regulated by TLR-mediated blood amyloid A protein stimulation and lysosome rupture, which stimulate the formation of the leucine-rich repeat Nod-like receptor (NLR) family pyrin domain containing 3 (NLRP3) (Fig. 4). This step in turn results in the activation of the caspase family, causing cell apoptosis, changes in the permeability of endothelial cells, IL1-β release, and IL-18 release. CTSB^*–/–*^ and CTSB inhibition with a pharmacological inhibitor of CTSB, or with short interfering RNA led to a suppression of pro-TNF-α secretion and its intracellular accumulation in response to lipopolysaccharides [101], suggesting that CTSB plays a role in pro-TNF-α maturation. In 2017, a large scale clinical trial showed an essential role of IL-1β in cardiovascular diseases [102]. Cholesterol crystals have been shown to stimulate NLRP3-mediated production of IL-1β, a process that requires CTSL and CTSB (Fig. 4) [94]. Serum amyloid A (SAA) can also induce NLRP3-mediated IL1β expression via the TLR-2/4 signaling pathways [103]. It was reported that both the NLRP3 inflammasome adaptor molecule apoptosis-associated speck-like protein containing a CARD (ASC) and CTSB were involved in the subsequent release of mature IL-1β (Fig. 4) [94]. NLRP3 silencing as well as CTSB inhibition suppressed the IL-1β secretion induced by SAA in ASC^−/−^ macrophages [103], indicating activation of the CTSB-mediated NLRP3 inflammasome. SAA has been shown to activate the release of lysosomal CTSB into the intracellular space. Similarly, in the endothelial cells, palmitate resulted in caspase-1 activation via formation of NLRP3 inflammasome complex and IL-1β secretion, which led to a lowering of the expression of the EC tight junction molecules ZO1 and ZO2 and an enhancement of EC permeability; these changes were rectified by three types of inhibitors, i.e., the CTSB inhibitor CA074Me, an inflammasome inhibitor (YVAD), and a high mobility group protein B1 inhibitor (glycyrrhizin) [104]. Moreover, Murphy et al. demonstrated that amyloid-β40 and amyloid-β42 enhanced cytosol CTSB/CTSL activity, caspase-3/-9 activation, and pro-IL-1β activation and secretion to the extracellular space [105]. In the same paper, the authors revealed that CTSL and CTSB also degraded NLRP10 (which is known as a negative regulator of the NLRP3 inflammasome), which triggered the formation of the NLRP3 inflammasome complex (Fig. 4) [105]. In addition, in macrophages, CTS inhibition by CA047Me and cystatin C/B (endogenous CTS inhibitors) exerted a beneficial effect on sterile particle-induced IL-1β secretion [106]. It is notable that although multiple CTSs participate in the synthesis and maturation of pro-IL-1β, which is associated with the NLRP3 inflammasome, CTS deletion studies revealed that individual CTSs exhibited quite different efficacy in promotion of these events [106]. Thus the role of the increased individual CTSs in NLRP3 inflammasome activation and IL-1β production/maturation in cardiovascular cells and inflammatory cells may be more complicated than our current knowledge suggests.

*T-cell activation*: T-cell activation modulates the inception and progression of ACVDs depending on the type of T cells and the particular ACVD. There are limited studies on the interactions between CTSs and T-cells in ACVDs. CTSL, CTSF, and CTSS participate in antigen presentation in professional antigen-presenting cells (APCs), macrophages, and thymic epithelial cells by processing the major histocompatibility complex class (MHC)-II-associated invariant chain (Ii; CD74), respectively [107]. In 1999, it was reported that CTSS is a key player in the processing of MHC-II peptide in splenocytes and dendritic cells [108]. A later study documented that CTSS but not CTSL can cleave Ii peptide in nonprofessional APCs [109]. A recent study demonstrated that CTSS-triggered antigen release induces the endosomal escape of the antigen, leading to increased MHC-I presentation in bone marrow-derived dendric cells [110]. CTSS^−/−^ reduces lymphoma growth by limiting communication with CD4^+^ T follicular helper cells while inducing antigen diversification and activation of CD8^+^ T cells [111]. Moreover, CTSS inhibition with its selective inhibitor (JNJ-39641160) interrupted T-cell activation and cytokine releases [112]. In bone marrow-derived macrophages, interferon-γ-mediated MHC-II presentation is regulated by CTSS but not CTSL [113]. In dendritic cells, IL-6 lowered intracellular MHC-II levels by STAT3 inactivation, leading to an increase in CTSS activity [114]. In the same study, the authors showed that CTSS overexpression lowered MHC-II levels and inactivated CD4^+^ T cells, and these alterations were rescued by the CTSS inhibitors. These findings raised the possibility that CTSS-mediated CD4^+^ T-cell maturation might be driven by STAT3 activation induced by different inflammatory cytokines. Conversely, CTSL specifically degraded Ii in cortical thymic epithelial cells [115]. In the same report, CTSL^−/−^ disturbed CD4^+^ T-cell-positive selection due to a defect of human leukocyte antigen (HLA)-DR isotype presentation and Ii processing in thymic epithelial cells [115]. Pharmacological CTSL inhibition was found to lower CD8^+^ T-cell activity by inactivation of CTSC, which is required for T-cell pro-granzyme activation [116]. Of note, mice have only CTSL but humans have both CTSL and CTSL2 (also called CTSV) [3]. Similar to CTSL, CTSV helps in MHC-II molecule loading and in antigen generation in thymic epithelial cells [117]. In addition to Ii degradation, several CTSs also modulate antigenic peptide production. The presence of CTSH and CTSB resulted in the production of antigenic peptide from influenza hemagglutinin H5N1-HA and type II collagen [118]. CTSK is required in bone marrow T-cell homeostasis [119]. CTSL has been demonstrated to produce chimeric epitopes via transpeptidation in diabetogenic CD4^+^ T-cell activation. How these CTSs activate T-cell, antigen production, and T-cell homeostasis in patients with ACVDs at the molecular level remains largely unknown.

*TLR-7 and TLR-9 processing-dependent and -independent modulation of effector and regulatory T-cell biology*: Recent evidence has drawn significant attention to the exploration of CTS-mediated T-cell activation, which drives the close relationship between autoimmune diseases and ACVDs [107]. CTSK inhibition promoted resistance to autoimmune arthritis and encephalomyelitis in mice, in conjunction with a lack of pathogen (DNA)-induced TLR9 signaling of APCs, which led to lowered T helper 17 (Th17) cell production without influencing the APC antigen presentation ability (Fig. 4, right) [120]. Cleavage of TLR7 has been demonstrated to be accomplished by furin peptidases in addition to cathepsins and asparagine endopeptidases [121]. In vivo and in vitro experiments revealed that in mice with lupus autoimmunity, CTSK partially contributed to the pathology of TLR-7 proteolytic fabrication and the consequent CD4^+^CD25^+^Foxp3^+^ regulatory T-cell biology (immunosuppressive activity, lifespan, and differentiation) [122]. CTSK has been shown to be involved in the development of psoriasis-like skin lesions through TLR-dependent Th17 activation in adjuvant-induced arthritis in rats [123]. Overexpression of CTSS exacerbates lupus pathogenesis through upregulation of TLR7 and IFN-α in transgenic mice [124]. Thus, both CTSS and CTSK function in TLR-7/-9 processing, which may also contribute to the development of autoimmune-related ACVDs. CTSL drives the differentiation of CD4^+^ T cells into T-helper-17 cells, and this effect was suppressed by a small CTSL-selective inhibitor, suggesting that targeting the CTSL regulatory module could be an approach to the management of Th17 cell-driven autoimmune disorders [125]. These observations add another layer to the growing evidence of CTS contributions to the activation of effector and regulatory T-cell biology. Given that genetic and pharmacological interventions of these CTSs exerted a beneficial effect on autoimmune diseases in model mice [120, 122], therapy with small CTS inhibitors might help lower the incidence and mortality of ACVD patients with and without autoimmune disease. Further clinical trials are necessary to explore this issue.

*Migration, proliferation and apoptosis*: Blood inflammatory cells (monocytes and leukocytes) and transmigration through the endothelium and basement membrane are essential pathological events in many AVDs. Vascular cell migration also contributes to cardiovascular wall remodeling during the initiation and progression of atherosclerotic lesions (Fig. 2A). CTSs, including CTSS, CTSK, CTSL, CTSC, and CTSZ, have been shown to participate in inflammatory and vascular cell adhesion and/or migration [48]. Moreover, vascular cell proliferation and apoptosis/death promote atherosclerotic lesion formation and its complications. There is accumulating evidence that CTS functions in inflammatory and vascular cell proliferation and apoptosis [4, 9]. The individual CTS cellular function in various ACVDs has been covered by a previous comprehensive review [21].

*ECM degradation*: ECM macromolecules (e.g., collagen, elastin, fibronectin, proteoglycans, and laminin) are essential components of the vascular wall. Unexpected changes in the synthesis and degradation of these ECM components trigger ACVD initiation and progression in humans and animals. The roles of CTS family members in ECM metabolism during ACVD development have been covered by a previous comprehensive review [21]. Accumulating evidence indicates that ACVD-related vascular cell and inflammatory cell events (adhesion, transmigration, proliferation, differentiation, apoptosis, microtubulogenesis, and antigen presentation) depend on the CTS-mediated intracellular and extracellular protein metabolism during ACVD. Based on the above-described findings, we propose that the increased lysosomal and nonlysosomal CTSs function collaboratively and/or independently as mechanisms to facilitate the development of ACVDs.

### Circulating CTSs as prognostic and diagnostic biomarkers for ACVDs

The potential of CTSs as prognostic cardiovascular disease biomarkers has been highlighted in several studies in the literature [14–16]. For example, CTSS has recently been associated with increased risk of all-cause mortality and ACVD death in patients with non-ST-segment elevation acute coronary syndromes [16]. In patients with stable coronary heart disease, CTSB is associated with high risk for cardiovascular events [15], arterial stiffening and atherosclerotic vascular disease [14]. CTSD is associated with heart failure severity and poorer outcome [126]. In addition, the altered levels of CTSs and their endogenous inhibitors in patients with ACVD, aneurysm, obesity, diabetes mellitus, and peripheral arterial disease indicate that these molecules can also serve as diagnostic biomarkers of ACVDs [2, 13]. A population-based clinical investigation demonstrated that elevated circulating cystatin C concentrations were linked to left ventricular hypertrophy and diastolic dysfunction [127]. We observed that increased levels of CTSK were associated with the presence of chronic heart failure [18]. We also found that circulating CTSK levels were increased in ACVD patients and were positively correlated with the plaque size and high-sensitive C-reactive protein levels but were negatively correlated with high-density lipoprotein cholesterol levels [17]. Blood CTSK levels were reported to be much higher in patients with persistent atrial fibrillation compared to those of patients with paroxysmal atrial fibrillation [128]. Moreover, circulating CTSK levels were positively correlated with the coronary artery calcification score in patients without diabetes mellitus [129]. Likewise, patients who had experienced an acute myocardial infarction had markedly higher plasma CTSK levels compared to both control subjects and patients with stable or unstable angina [130]. As expected, independent studies showed that patients with coronary artery disease had elevated levels of plasma CTSK, CTSS, CTSL, and CTSB compared to healthy individuals [17]. Recent studies showed that CTSK and CTSS levels were increased in blood and atherosclerotic plaques of mice under pathological stress conditions [131]. Thus, these data indicate that increased plasma CTSs serve as potential biomarkers of ACVD and therapeutic efficacy. It should be noted that there are as yet no confirmed data regarding the blood levels of single CTSs as specific predictive markers for patients with ACVD, since CTSs present no tissue- or cell-specific expression patterns. Large-scale clinical cohort investigations are required to determine whether increased circulating individual CTSs can serve as prognostic and/or diagnostic biomarkers in patients with ACVD.

### Therapeutics for CTSs in ACVDs

Cardiovascular drugs exert a pleiotropic effect that targets CTSs as part of the cellular signals and proteolytic pathway [2]. Inflammation-related factors (including cytokines, oxidative stress, and renin-angiotensin signaling) regulate CTS expression/activation, leading to the initiation and progression of ACVDs [5, 6]. Accumulating experimental evidence in animal models has documented that lipid-lowering statins and angiotensin II receptor blockers mitigated diet-induced atherosclerotic plaque growth and hypertensive cardiovascular-renal remodeling through inflammation- and oxidative stress-induced CTS expression and activity [56]. We observed that chronic stress accelerated atherosclerotic plaque growth and thrombosis, accompanied by increased CTSK and CTSK expressions; these changes were rectified by dipeptidyl peptidase 4 inhibition and glucagon-like peptide-1 agonists [59, 60]. Although there are only limited laboratory findings, the data that are available from clinical trials favor the notion that CTSS and CTSK may be potential molecular targets for the management of ACVDs. Collectively, the above findings emphasize the importance of determining whether CTS inhibitor treatment should be part of the best current management of ACVDs. Indeed, the evidence from several clinical studies indicates that systemic CTS inhibition can elicit off-target effects in patients with non-atherosclerotic diseases [132]. That might be why there are still no available drugs to target CTSs. However, the delivery of CTS inhibitors to specific tissues could be a promising future approach.

### CTSs as a diagnostic imaging tool in ACVDs

Several CTSs have gained attention as potential therapeutic targets in patients with ACVD based on the analysis of atherosclerosis using fluorescent probes, nanoparticles, and radiolabeled inhibitors. It was first reported in 2002 that CTSB imaging beacons were abundantly accumulated in activated macrophages of advanced plaques [133]. A decade later, the combination of CTSB (ProSense) and αvβ3 integrin (IntegriSense) near-infrared fluorescence (NIRF) agents was applied to the longitudinal capture of atherosclerotic lesions; in the same study, the CTSB NIRF agents were shown to be useful for monitoring ezetimibe-mediated anti-inflammation, for the preclinical testing of therapeutics, and potentially for the early diagnosis of patients with ACVD [134]. Two preclinical studies have shown that photodynamic therapy guided by CTSB and CTSS activities is an efficient means of lowering vascular inflammation and atherosclerotic plaque growth, suggesting that this approach could potentially be advanced in clinical trails [135, 136]. Over the past decade, there have been an increasing number of experimental and clinical molecular imaging studies using both CTSS and CTSB NIRF probes with their specific inhibitors for verification of the specificity of probes and individual proteases in atherosclerosis, cardiovascular inflammation, and chronic kidney disease [137, 138]. A 2017 study using intravascular fluorescence imaging assessed the everolimus-eluting stent-mediated stabilization of plaque inflammation [139]. A recent comprehensive review highlighted new intravascular NIRF imaging targets including macrophages, CTS proteolytic activity, oxLDL, and abnormal endothelial permeability [140]. It is notable that CTS-mediated NIRF probes have also been applied to other inflammatory disorders and tumor molecular imaging. Collectively, these data raise the possibility that an endovascular approach with an active CTS sensor could overcome the limited depth penetration of NIRF probes to assist in the clinical detection of deep and complicated atherosclerotic plaques. Advanced technology with CTS-specific NIRF probes could be used to evaluate the clinical applications of enhanced tissue CTS activity, with the ultimate goal of developing a more effective noninvasive approach to diagnosing ACVDs and predicting their prognosis.

## Conclusions and perspectives

Following the identification of members of the human CTS family, numerous human and animal pathology studies, biomarker and imaging studies, and sophisticated mechanistic and biochemical investigations were performed and revealed multiple divergent roles for individual CTSs in ACVDs and their complications. It has recently become clear that CTSs function not only as simple housekeeping enzymes in lysosomes, but also in essential capacities outside of the lysosomes (e.g., in the extracellular space, nucleus, plasma membrane, and cytosol), including as regulatory proteases. Laboratory studies of knockout and transgenic mice have provided direct evidence that increased CTSs are involved in ACVDs through the intracellular signal cascades, receptor activation, modifications of angiogenic growth factors, cytokines, and HDAC-6, lipid metabolism, cell events (apoptosis and proliferation), angiogenesis, and matrix protein remodeling. Several safe and highly selective CTS inhibitors have been developed in animals and humans, but no CTS inhibitor has become available for ACVD or other diseases since the first identifications of human CTSS and CTSK nearly 30 years ago. Accumulating evidence suggests that CTSs that are increased in plasma and local tissue can be applied as diagnostic tools as well as biomarkers and imaging probes. Because these small compounds might also influence the mechanisms underlying ACVDs, their efficacy against ACVDs must also be evaluated. In addition, because the prevalence of ischemic peripheral and coronary artery diseases associated with the various psychological stresses of modern life continue to increase in elderly populations, dual therapy targeting ACVDs and associated pathological stress should also be taken into account when treatment strategies are designed. Clinical trials with large patient series are necessary to determine whether reversible and selective CTS inhibitors can be safe and effective for the management of patients with ACVDs.

## Supplementary Information

**Additional file1**: **Table S1.** Different factors regulate cathepsin expression in cardiovascular system.

## Data Availability

All data are included in the manuscript.
