# Supplementary material for: Overview of multifunctional cysteinyl cathepsins in atherosclerosis-based cardiovascular disease: from insights into molecular functions to clinical implications
Source: Cell Biosci. 2023 May 19;13:91. doi: 10.1186/s13578-023-01040-4 (PMC10197855; doi:10.1186/s13578-023-01040-4)
Supplement: Supplementary file 1 — Additional file1: Table S1. Different factors regulate cathepsin expression in cardiovascular system. [file 13578_2023_1040_MOESM1_ESM.pdf]

Suppl. Table S1. Different factors regulate cathepsin expression in cardiovascular system

Target cells	IL-8, Insulin-like growth factor II receptor	Target cathepsins (CTSs)	Expression change	Refs.
Endothelial cells	PPAR-α agonist Wy14643	Cathepsin B	Increase	1, 2
	Advanced glycation end products	Cathepsin B	Decrease	3
	FGF2, IFN-γ, IL-1β, IL-6, LPS, TNF-α	Cathepsin D	Decrease	4
	Oscillatory shear stress	Cathepsins S, K, L	Increase	5, 6
	FGF2, IFN-γ, IL-1β, TNF-α	Cathepsins K, L	Increase	6, 7
Vascular smooth muscle	TIN-ag-RP	Cathepsins L, S	Increase	5, 8
	Advanced glycation end products	Cathepsin B	Decrease	9
	CpG ODNs, IFN-γ, IL-1β, IL-6, LPS, TNF-α	Cathepsin D	Decrease	10
Macrophages	Angiotensin II	Cathepsins S, K, L	Increase	5,11,12
	Serum from patients with rheumatoid arthritis	Cathepsin F	Increase	13
	Interferon-c	Cathepsin B	Increase	14
	Oxidized LDL cholesterol	Cathepsin B	Increase	15
	Amyloid-β_{40}, amyloid-β_{42}	Cathepsins S, K	Increase	16, 17
	TNF-α	Cathepsin S	Increase	18
	IL-4, IL-10, IL-13	Cathepsin S	Increase	19
	Oscillatory shear stress	Cathepsin K	Decrease	20
Neutrophils	2',3-dihydroxy-5-methoxybiphenyl	Cathepsin B	Increase	21
	PPAR-γ	Cathepsin G	Decrease	22

Abbreviations: CpG ODN, CpG oligodeoxynucleotide; FGF2, fibroblast growth factor 2; IFN-γ, interferon-gamma; LDL, low-density lipoprotein; IL, Interleukin; LPS, lipopolysaccharide; PPAR, peroxisome proliferator-activated receptor; TNF-α, tumor necrosis factor-alpha.

References

1. Rose PP, Bogyo M, Moses AV and Fruh K. Insulin-like growth factor II receptor-mediated intracellular retention of cathepsin B is essential for transformation of endothelial cells by Kaposi's sarcoma-associated herpesvirus. *Journal of virology*. 2007;81:8050-62.
2. Schraufstatter IU, Trieu K, Zhao M, Rose DM, Terkeltaub RA and Burger M. IL-8-mediated cell migration in endothelial cells depends on cathepsin B activity and transactivation of the epidermal growth factor receptor. *Journal of immunology* (Baltimore, Md : 1950). 2003;171:6714-22.
3. Reichenbach G, Starzinski-Powitz A, Sloane BF, Doll M, Kippenberger S, Bernd A, Kaufmann R and Meissner M. PPARalpha agonist Wy14643 suppresses cathepsin B in human endothelial cells via transcriptional, post-transcriptional and post-translational mechanisms. *Angiogenesis*. 2013;16:223-33.
4. Li Y, Chang Y, Ye N, Dai D, Chen Y, Zhang N, Sun G and Sun Y. Advanced Glycation End Products Inhibit the Proliferation of Human Umbilical Vein Endothelial Cells by Inhibiting Cathepsin D. *International journal of molecular sciences*. 2017;18.
5. Liu J, Sukhova GK, Yang JT, Sun JS, Ma LK, Ren A, Xu WH, Fu HX, Dolganov GM, Hu CC, Libby P and Shi GP. Cathepsin L expression and regulation in human abdominal aortic aneurysm, atherosclerosis, and vascular cells. *Atherosclerosis*. 2006;184:302-311.
6. Platt MO, Ankeny R and Jo HJ. Laminar shear stress inhibits cathepsin L activity in endothelial cells. Arterioscl Throm Vas. 2006;26:E89-E89.
7. Platt MO, Ankeny RF, Shi GP, Weiss D, Vega JD, Taylor WR and Jo H. Expression of cathepsin K is regulated by shear stress in cultured endothelial cells and is increased in endothelium in human atherosclerosis. *American journal of physiology Heart and circulatory physiology*. 2007;292:H1479-86.
8. Sukhova GK, Shi GP, Simon DI, Chapman HA and Libby P. Expression of the elastolytic cathepsins S and K in human atheroma and regulation of their production in smooth muscle cells. *Journal of Clinical Investigation*. 1998;102:576-583.
9. Wex T, Lipyansky A, Bromme NC, Wex H, Guan XQ and Bromme D. TIN-ag-RP, a novel catalytically inactive cathepsin B-related protein with EGF domains, is predominantly expressed in vascular smooth muscle cells. *Biochemistry*. 2001;40:1350-7.

10. Ma M, Guo X, Chang Y, Li C, Meng X, Li S, Du ZX, Wang HQ and Sun Y. Advanced glycation end products promote proliferation and suppress autophagy via reduction of Cathepsin D in rat vascular smooth muscle cells. *Molecular and cellular biochemistry*. 2015;403:73-83.
11. Kitamura H, Kamon H, Sawa S, Park SJ, Katunuma N, Ishihara K, Murakami M and Hirano T. IL-6-STAT3 controls intracellular MHC class II alphabeta dimer level through cathepsin S activity in dendritic cells. *Immunity*. 2005;23:491-502.
12. Cheng XW, Kuzuya M, Nakamura K, Di Q, Liu ZX, Sasaki T, Kanda S, Jin H, Shi GP, Murohara T, Yokota M and Iguchi A. Localization of cysteine protease, cathepsin S, to the surface of vascular smooth muscle cells by association with integrin alpha nu beta 3. *American Journal of Pathology*. 2006;168:685-694.
13. Kaakinen R, Lindstedt KA, Sneck M, Kovanen PT and Oorni K. Angiotensin II increases expression and secretion of cathepsin F in cultured human monocyte-derived macrophages: an angiotensin II type 2 receptor-mediated effect. *Atherosclerosis*. 2007;192:323-7.
14. Ostensen M, Morland B and Husby G. Stimulation of murine macrophage cathepsin B by serum from patients with rheumatoid arthritis: an indicator of disease activity. *Clinical and experimental immunology*. 1983;51:103-9.
15. Li Q, Ding L and Bever CT, Jr. Interferon-c induces cathepsin B expression in a human macrophage-like cell line by increasing both transcription and mRNA stability. *International journal of molecular medicine*. 1998;2:181-186.
16. Burns-Kurtis CL, Olzinski AR, Needle S, Fox JH, Capper EA, Kelly FM, McQueney MS and Romanic AM. Cathepsin S expression is up-regulated following balloon angioplasty in the hypercholesterolemic rabbit. *Cardiovascular research*. 2004;62:610-620.
17. Vasiljeva O, Papazoglou A, Kruger A, Brodoefel H, Korovin M, Deussing J, Augustin N, Nielsen BS, Almholt K, Bogyo M, Peters C and Reinheckel T. Tumor cell-derived and macrophage-derived cathepsin B promotes progression and lung metastasis of mammary cancer. *Cancer research*. 2006;66:5242-50.
18. Castellano J, Badimon L and Llorente-Cortes V. Amyloid-beta Increases Metallo- and Cysteine Protease Activities in Human Macrophages. *J Vasc Res*. 2014;51:58-67.
19. Lindahl C, Simonsson M, Bergh A, Thysell E, Antti H, Sund M and Wikstrom P. Increased levels of macrophage-secreted cathepsin S during prostate cancer progression in TRAMP mice and patients. *Cancer genomics & proteomics*. 2009;6:149-59.

20. Lugering N, Kucharzik T, Stein H, Winde G, Lugering A, Hasilik A, Domschke W and Stoll R. IL-10 synergizes with IL-4 and IL-13 in inhibiting lysosomal enzyme secretion by human monocytes and lamina propria mononuclear cells from patients with inflammatory bowel disease. *Dig Dis Sci*. 1998;43:706-14.
21. Akenhead ML, Fukuda S, Schmid-Schonbein GW and Shin HY. Fluid shear-induced cathepsin B release in the control of Mac1-dependent neutrophil adhesion. *Journal of leukocyte biology*. 2017;102:4-126.
22. Liao HR, Chen IS, Liu FC, Lin SZ and Tseng CP. 2',3-dihydroxy-5-methoxybiphenyl suppresses fMLP-induced superoxide anion production and cathepsin G release by targeting the beta-subunit of G-protein in human neutrophils. *European journal of pharmacology*. 2018;829:26-37..
